# Transfer of Patients with Spontaneous Intracranial Hemorrhage who Need External Ventricular Drain: Does Admission Location Matter?

**DOI:** 10.5811/westjem.2020.10.47795

**Published:** 2021-01-12

**Authors:** Quincy K. Tran, Sagar Dave, Daniel J. Haase, Laura Tiffany, Shannon Gaasch, Wan-tsu W. Chang, Kevin Jones, Matthew J. Kole, Aaron Wessell, Gary Schwartzbauer, Thomas M. Scalea, Jay Menaker

**Affiliations:** * University of Maryland School of Medicine, Department of Emergency Medicine, Baltimore, Maryland; † University of Maryland School of Medicine, The R. Adams Cowley Shock Trauma Center, Baltimore, Maryland; ‡ University of Maryland Medical Center, Department of Surgical Critical Care, Baltimore, Maryland; § University of Maryland School of Medicine, Department of Neurosurgery, Baltimore, Maryland; ¶ University of Maryland School of Medicine, Department of Surgery, Baltimore, Maryland

## Abstract

**Introduction:**

Patients with spontaneous intracranial hemorrhage (sICH) are associated with high mortality and require early neurosurgical interventions. At our academic referral center, the neurocritical care unit (NCCU) receives patients directly from referring facilities. However, when no NCCU bed is immediately available, patients are initially admitted to the critical care resuscitation unit (CCRU). We hypothesized that the CCRU expedites transfer of sICH patients and facilitates timely external ventricular drain (EVD) placement comparable to the NCCU.

**Methods:**

This is a pre-post study of adult patients transferred with sICH and EVD placement. Patients admitted between January 2011–July 2013 (2011 Control) were compared with patients admitted either to the CCRU or the NCCU (2013 Control) between August 2013–September 2015. The primary outcome was time interval from arrival at any intensive care units (ICU) to time of EVD placement (ARR-EVD). Secondary outcomes included time interval from emergency department transfer request to arrival, and in-hospital mortality. We assessed clinical association by multivariable logistic regressions.

**Results:**

We analyzed 259 sICH patients who received EVDs: 123 (48%) CCRU; 81 (31%) 2011 Control; and 55 (21%) in the 2013 Control. The groups had similar characteristics, age, disease severity, and mortality. Median ARR-EVD time was 170 minutes [106–311] for CCRU patients; 241 minutes [152–490] (p < 0.01) for 2011 Control; and 210 minutes [139–574], p = 0.28) for 2013 Control. Median transfer request-arrival time for CCRU patients was significantly less than both control groups. Multivariable logistic regression showed each minute delay in ARR-EVD was associated with 0.03% increased likelihood of death (odds ratio 1.0003, 95% confidence interval, 1.0001–1.006, p = 0.043).

**Conclusion:**

Patients admitted to the CCRU had shorter transfer times when compared to patients admitted directly to other ICUs. Compared to the specialty NCCU, the CCRU had similar time interval from arrival to EVD placement. A resuscitation unit like the CCRU can complement the specialty unit NCCU in caring for patients with sICH who require EVDs.

## INTRODUCTION

Spontaneous intracranial hemorrhage (sICH) is associated with up to 40% mortality.^1^,^2^ External ventricular drain (EVD) placement in these patients has been associated with improved mortality and functional outcomes.^3^–^5^ When patients with sICH present to an emergency department (ED) that does not have neurosurgical consultation capabilities, they are typically transferred to a tertiary care center for further evaluation and management. However, the process for non-trauma patient transfer has been fragmented,^6^ resulting in a significant delay for these critically ill patients.^7^ As a result, delays in the transfer of patients with sICH would result in worse outcomes.^8^,^9^

At our urban, academic, tertiary care center, the neurocritical care unit (NCCU) is the preferable unit to receive sICH patients, especially those in need of EVD placement, from referring facilities. Historically, when there was no immediately available bed in the NCCU, patients were transferred to any adult intensive care unit (ICU) with an available bed. However, in an effort to streamline the process of transfer of patients with critical illnesses or time-sensitive disease (Type A aortic dissection, ischemic stroke, sICH, etc), our institution established the critical care resuscitation unit in July 2013. The CCRU is a six-bed ICU-based resuscitation unit that is staffed by a 24/7 team of intensivists and advanced practice providers (APP). It focuses on rapid transfer of critically ill patients or patients with time-sensitive diseases for initial resuscitation and evaluation before transferring them to a specialized ICU.^10^ For patients with sICH, when there are no available beds at the NCCU, the CCRU admits, provides initial resuscitation, manages blood pressure, and supports for EVD placement as indicated. Once stabilized, patients are subsequently transferred to an available bed at the NCCU for further longitudinal care.

The CCRU represents an alternative admission location for patients with sICH, but its effectiveness in caring for these patients is not known. In this study we aimed to investigate the CCRU’s efficacy in caring for patients with sICH who required EVD placement. We hypothesized that the CCRU would expedite the transfer of sICH patients and provide comparable care to the subspecialty NCCU, including timely EVD placement. We also investigated whether timely placement of EVD would be associated with outcomes of sICH patients with suspected elevated intracranial pressure (ICP).

## METHODS

### Study Settings

We performed a retrospective pre-post chart review of adult patients sustaining sICH who were transferred to our academic medical center and received EVD after arrival. We included patients who were transferred from any referring EDs between January 1, 2011–September 30, 2015.

Our academic tertiary care center has a neurosurgical residency. Neurosurgery residents and senior residents provide coverage at our medical center around the clock. The residents evaluate patients with intracranial hemorrhage when they first arrive at our medical center. Once the neurosurgery team decides whether EVD placement is indicated, one of the residents will insert the EVD at the appropriate ICU. Therefore, for patients who have signs and symptoms of significant intracranial hypertension, the sooner the patients arrive at our institution, the sooner they will undergo this life-saving procedure. Furthermore, there has not been any change in the coverage of neurosurgical residents during our study period.

**Population Health Research Capsule**

What do we already know about this issue?

Spontaneous intracranial hemorrhage and intracranial hypertension are associated with high mortality and require early neurosurgical interventions.

What was the research question?

Does the critical care resuscitation unit (CCRU) expedite transfer and facilitate early intervention? Does time interval to external ventricular drain (EVD) placement matter?

What was the major finding of the study?

The CCRU decreased time to EVD placements. Delayed EVD placement was associated with higher mortality.

How does this improve population health?

A CCRU can complement the neurocritical care unit to improve outcomes by reducing emergency department transfer delays; facilitating similar time to EVD placements.

Clinicians from other facilities refer their patients to our medical center via our 24/7 in-hospital centralized center, Express Care. For example, the referring clinician first makes a request transfer to our transfer center. The Express Care staff then connects the referring clinician to the on-call neurosurgeon and the NCCU physician. When the patient is considered to need immediate transfer but there is no available NCCU bed, the CCRU attending physician will be contacted for bed request. This transfer process is uniform for all patient transfers from referring hospitals to any inpatient unit at our medical center.

This study was approved by our institutional review board.

### Patient Selection

We queried our academic center’s electronic health records (EHR) to identify eligible patients. Patients were identified by *International Classification of Diseases*, ninth revision (ICD-9 codes of 430.XX, 431.XX) for any sICH and procedure code 02.21 for EVD.^11^,^12^ Patients who had sICH and were transferred directly from other hospitals’ EDs were eligible.

We selected three groups of patients with sICH and EVD placement for comparison. The first group (CCRU) included patients who were admitted initially to the CCRU between August 1, 2013–September 30, 2015. The second group included a historical cohort of sICH patients (2011 Control) who were admitted to any adult ICU between January 1, 2011–July 31, 2013, before the CCRU opening. The third group (2013 Control) contained patients who were directly admitted to the NCCU during the same period when the CCRU became operational (August 1, 2013–September 30, 2015). We included this group for comparison because when the NCCU has an available bed, it can bypass the CCRU and admit a patient directly from any ED. This direct admission should be associated with shorter transfer delay.

We excluded trauma patients and patients whose hemorrhage was due to secondary pathologies, such as tumor, arteriovenous malformations, or ischemic stroke, because the common neurosurgical severity scores were neither designed for nor validated in these patients.^13^,^14^ We excluded patients who did not have sufficient records or patients who did not have documentation of EVD after arrival at our academic center. Patients who presented first to our academic center’s ED were also excluded. These patients did not have to undergo the transfer process between hospitals, which had been associated with delays of care. Moreover, they usually had early access to interventions by neurosurgical teams at our institutions. As a result, these patients would have different outcomes from patients who were transferred from other hospitals. Furthermore, the sample size for this group was small and would not provide meaningful statistical analysis.

### Outcomes

The primary outcome was the time intervals from arrival at the CCRU or our ICUs to the time of EVD placement. Secondary outcomes included a) time intervals from transfer request to arrival at one of our medical center’s ICUs; and b) in-hospital all-cause mortality.

### Data Collection

The principal investigator (PI) of the study trained the other investigators who were not blinded to our hypothesis to extract data from patients’ records into a standardized Microsoft Access database (Microsoft Corp., Redmond, WA). Investigators input data in sections and independently of each other to reduce bias. For example, investigators who collected data for disease severity did not have access to data regarding patients’ EVD placement or outcomes. The disease severity scores for subarachnoid hemorrhage (SAH) were the Hunt and Hess Scale [H&HS], and the World Federation of Neurological Surgeons Scale [WFNSS]. The severity scores for patients with spontaneous intraparenchymal hemorrhage were the Intracerebral Hemorrhage Score [ICHS], and the Functional Outcome in Patients with Primary Intracerebral Hemorrhage scale [FUNC score]. Up to 20% of the data (time interval to EVD placement, ICP measurements) was independently validated by another investigator to maintain at least 90% inter-rater agreement. Discrepancies were adjudicated by the PI during the group’s quality meetings every three months, until data extraction was completed.

Patient data were obtained from multiple sources including patients’ ED records, the accepting ICUs’ flow sheets and our institution’s EHR. Time of EVD placements and ICP measurements were obtained from procedural notes and nursing notes. Time of arrival at the NCCU or CCRU, and patient mortality were obtained from our EHR.

### Data Analysis

We used descriptive analyses (mean ± standard deviation [SD], median (interquartile ranges [IQR]), and number [n] [%]) for demographic and clinical factors to compare groups. Continuous data between two groups were analyzed via Student’s t-test or the Mann-Whitney tests, as appropriate. We analyzed continuous data between three groups via analysis of variance with Holm-Sidak post-hoc test or Kruskal-Wallis with Dunn’s post-hoc tests. We compared categorical data by chi-square test. We used the Kaplan-Meier graph to present the time interval between ICU arrival and EVD placement, and time interval between transfer request and arrival at the CCRU or other ICUs. Any event that occurred after six hours was reported and analyzed as occurring after six hours from the index time.

We performed multivariable logistic regression to assess associations between clinical variables and in-hospital all-cause mortality. Prior to analysis, we visually inspected the histograms of the time intervals between arrival-EVD placement and transfer request to ICU arrival for their patterns of distribution. Based on their patterns of distribution, no transformation was necessary. To identify relevant independent variables for the multivariable logistic regressions, we first performed univariable logistic regression using single independent variable and mortality. We included a priori-determined clinically significant factors (admitting to the CCRU; Arr-EVD, transfer request to arrival), and any independent variable with *p*-value ≤ 0.10^15^,^16^ in the multivariable logistic regression. Goodness-of-fit of our regression was assessed using the Hosmer-Lemeshow test, for which a *p*-value > 0.05 was considered a good fit.

Since patients with SaH or intraparenchymal hemorrhage have different physiopathology and severity scores, we a priori decided during our planning sessions to perform subgroup analyses involving patients with SAH only and patients with intraparenchymal hemorrhage only to investigate the effect of time to EVD placement and outcomes in these particular groups. We performed separate logistic models, adjusting for appropriate disease severity, in subgroups of patients with SAH or intraparenchymal hemorrhage only, using *a priori-determined factors* as stated above. For example, in the multivariable logistic regression model for patients with SAH, we included only the H&HS and the WFNSS.

All two-tailed *p*-values of < 0.05 were considered statistically significant. We performed statistical analyses using Sigma Plot version 14 (Systat Software Inc., San Jose, CA).

## RESULTS

### Patient Characteristics

**Figure 1:**
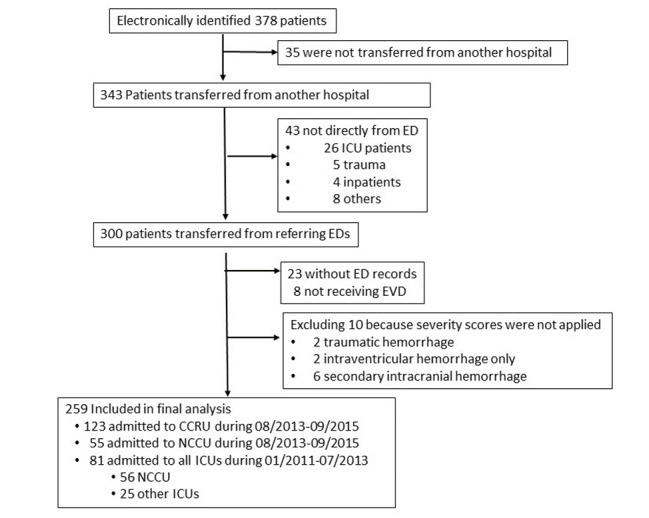
Patient selection diagram. *ED*, emergency department; *CCRU*, critical care resuscitation unit; *EVD*, external ventricular drain; *ICU*, intensive care unit; *NCCU*, neurocritical care unit.

We identified 343 patients who were transferred from other hospitals to our institution and received EVD placement between January 2011–September 2015. Of these, 259 patients who were transferred from various EDs met inclusion criteria and were included in the final analysis (Figure 1).

A total of 123 patients were admitted initially to the CCRU with an average of 4.9 patients per month from August 2013–September 2015. A total of 81 patients were transferred to various adult ICUs at our institution from January 2011–July 2013, with an average of 2.7 patients per month (Table 1A). Subsequently, 55 patients were admitted directly to the NCCU between August 2013–September 2015 with an average of 2.2 patients per month.

For patients with SAH, the median H&HS of patients admitted to the CCRU was 3 (IQR 2–4]. The median H&HS was 3 [2–4] and 2 [2–3] for 2011 Control patients and 2013 Control patients, respectively. The median WFNSS of patients admitted to the CCRU was 4 [2–4]. The median (IQR) WFNSS for 2011 Control patients (01/2011–07/2013) and 2013 Control (08/2013–09/2015) was 4 [2–5] and 2 [2–4], respectively. Other demographic characteristics between groups were similar (Table 1A).

Table 1B shows the demographic characteristics between patients with only SAH or only intraparenchymal hemorrhage. Invasive mechanical ventilation in EDs among patients who had intraparenchymal hemorrhage was more frequent (75%), compared to 47% (p = 0.001) among patients with SAH only. The opening ICP for patients with intraparenchymal hemorrhage was similar to those with SAH only (21 [8] centimeters water [cm H_2_0] vs 22 (7), p = 0.36) (Table 1B).

### Outcomes

**Figure 2:**
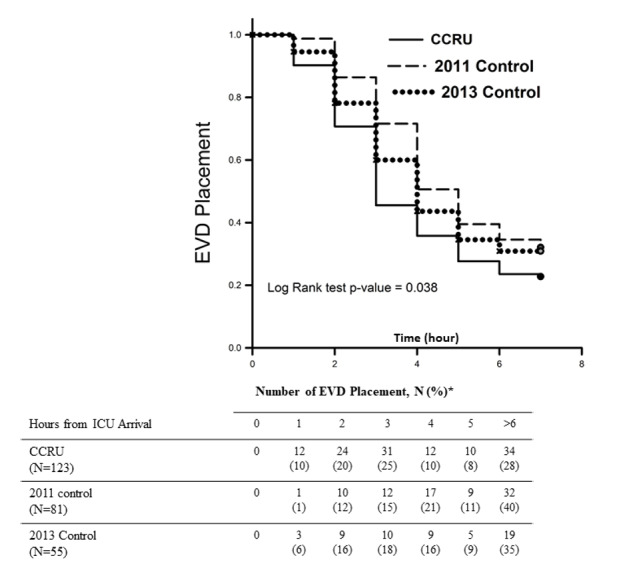
Comparisons of time intervals from arrival at intensive care unit to placement of external ventricular drain between critical care resuscitation unit, 2011 Control, 2013 Control groups. *percentage of EVD placement at a particular hour. *CCRU*, critical care resuscitation unit; *ICU*, intensive care unit; *EVD*, external ventricular drain.

Overall, the CCRU facilitated significantly earlier EVD placement for transferred patients with sICH when compared with the historical 2011 Control patients who were transferred between January 2011–July 2013 (Figure 2), but not the 2013 Control patients. The median time interval from arrival to EVD placement was 170 minutes [106–311] for patients admitted initially to the CCRU, compared to 241 minutes [152–490] (p < 0.01) for 2011 Control and 210 minutes [139–574], p = 0.28) for 2013 Control (Table 2A).

We performed a post-hoc analysis between the CCRU and the 2013 group to investigate whether the difference in time interval between arrival and EVD placement between the CCRU and the 2011 group was due to change in practice. We found that up to 54% of CCRU patients received EVD placement within three hours from CCRU arrival (Figure 2), while only 40% of the 2013 control group received EVD placement. This difference was statistically different by chi-square test (95% CI, 1.005–3.09, p = 0.047). This difference suggested that change of neurosurgery practice alone would not explain our findings, as this data was collected between two groups during the same period of time.

There were no significant different in time-to-event intervals between subgroup of patients with SAH only or patients with intraparenchymal hemorrhage only (Table 2B).

**Figure 3:**
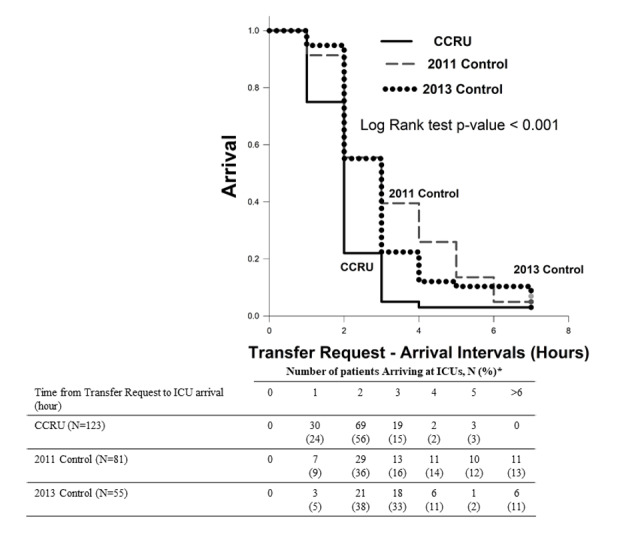
Comparisons of time interval from transfer request to arrival at intensive care units for critical care resuscitation unit patients, 2011 Control, 2013 Control groups. *percentage of patients arriving at the ICU at a particular hour. *CCRU*, critical are resuscitation unit; *ICU*, intensive care unit.

Time interval from transfer request to arrivals for patients initially admitted to the CCRU was 84 minutes [61–111], compared to 135 minutes [89–225] (p < 0.001) for 2011 Control and 132 minutes [99–177] (p < 0.001) for 2013 Control (Figure 3) and (Table 1B). Hospital outcomes (mortality, length of stay or rates of discharge home) in bivariate analyses were similar between patients who were transferred directly to the CCRU or other ICUs during different time periods (Table 2A). The percentage of discharge home for patients who had only intraparenchymal hemorrhage were significantly less than those with SAH only (9% v. 28%, p = 0.01) (Table 2B).

In our multivariable logistic regression for all patients, each minute longer from time interval between ICU arrival and EVD placement was associated with 0.03% of increased likelihood of death (odds ratio [OR] 1.0003, 95% CI, 1.0001–1.006, p = 0.043) (Table 3). In other words, each 30 minutes in delay of EVD placement was associated with 1% increased likelihood of death in our patient population. In the subgroup analysis of patients with SAH, the multivariable logistic regression, adjusting for disease severity for SAH was associated with higher likelihood of death (OR 1.0001, 95% CI, 1.0001–1.001, p = 0.016). However, time interval between ICU arrival and EVD placement was not significantly associated with mortality in the subgroup of patients with intraparenchymal hemorrhage (Table 2).

## DISCUSSION

Our study demonstrated that the CCRU contributed to more than a 200% increase in transfers of patients with sICH requiring EVD placement to our institution. However, this increase did not account for the number of patients who presented directly to our institution’s ED or those who were not transferred from a referring ED. Compared to the historical cohort, patients admitted to the CCRU experienced a shorter time interval from transfer request to arrival, and shorter time interval from arrival to EVD placement. Our study also suggested that longer time interval from arrival to EVD placement was associated with higher likelihood of mortality; however, more studies are needed to confirm our observation.

The CCRU at our academic medical center is a six-bed unit that was created to expedite a high volume of transfers and to provide timely resuscitation for critically ill patients, as described previously.^10^,^17^ It serves as a multidisciplinary unit to provide resuscitative efforts to all adult critically ill patients who need immediate resuscitation, whether the patients come from other facilities or from our own medical center. To achieve this purpose, the CCRU was specially designed to expedite transfer of patients who need immediate resuscitation.

To reduce unnecessary miscommunications and delays, a transfer request for patient to the CCRU involves the referring clinician, the specialty consultant attending, and the CCRU attending physician. During this phone conversation for transfer request, the CCRU attending physician initiates the transfer process immediately without having to wait for the request to officially appear on our institution’s bed tracking system, unlike other traditional ICUs at our medical center. Additionally, a plan of care for the patient before transfer, during transfer and upon arrival at the CCRU is proposed between the attending physicians once the patient is accepted for transfer to the CCRU. This anticipatory plan of care enables the CCRU team to prepare for necessary interventions prior to the patient’s arrival, including uncross-matched blood products, infusion medications at patient’s bedside, alerting operating rooms, mobilizing surgical teams, etc. Using this anticipatory plan allowed the CCRU to bring surgical patients to the operating rooms sooner than those who were historically admitted to other traditional ICUs.^17^

There are other potential reasons for the difference in time interval from arrival to EVD placement between the CCRU and other ICUs. The first reason could be a result from different volumes, as higher volume could be associated with higher efficiency. Furthermore, the CCRU’s nursing staff was designed to provide immediate resuscitation. The CCRU employs a flexible nursing model, so one to two nurses can be reassigned to assist with the resuscitative efforts for a critically ill patient, or a patient who would need an immediate life-saving procedure, without compromising care for other patients. This flexible nursing model, which allows the CCRU to maximize the efforts on patients’ resuscitations, is possible partly because CCRU nurses are not tied up with other chronic, longitudinal care as are nurses in traditional ICUs. Additionally, the CCRU attending physician is available 24 hours in the unit to provide immediate support for procedures, such as providing moderate sedation and airway management during EVD placement, while the APP provides care for other patients. Therefore, the CCRU team could provide fast and efficient support for our specialists to initiate life-saving procedures.

The design of the CCRU allows the unit to receive transfer of a wide variety of critically ill patients.^10^ Additionally, the staffing model and high volumes of transfer enable the CCRU to becomes more efficient in the immediate resuscitation of these patients. As a result of this efficiency, the resuscitation provided for these patients can be comparable to other specialty ICUs, as shown in this study with sICH patients. Once patients receive adequate resuscitation, they are transferred to the specialty ICUs where staff is well trained for longitudinal care. If there is no available bed once the patient is stabilized, the CCRU will continue to care for the patient until an available bed at an appropriate unit becomes available. To improve CCRU bed flow, patients from the CCRU would have the second highest priority after our own medical center’s ED patients, for the first available and appropriate bed. As a result, within a few hours of a patient’s arrival, another bed in the CCRU becomes available to receive the next critically ill patient(s). Therefore, the CCRU can complement the neurocritical care unit (NCCU) or other specialty ICUs to care for critically ill patients in the acute and hyperacute phase, while being able to reduce delays of transfer from referring EDs or from within our medical center.

Having the six-bed CCRU, or a similar resuscitation unit, is considered more efficient use of beds than opening up more beds in each of the six adult specialty ICUs at our medical center: cardiac surgical ICU, coronary care unit, medical ICU, NCCU, surgical ICU, and trauma ICU. Since the CCRU admits patients from all medical, surgical specialties and trauma,^10^,^17^ each adult specialty ICU would hypothetically need to create one extra ICU bed to accommodate these transfers, or the equivalent of the CCRU’s six beds. Furthermore, transfer requests for any single disease state are not uniformly distributed across time. Consequently, the NCCU, for example, would have to keep an open ICU bed while there is no patient requiring immediate EVD placement. On the other hand, the CCRU can use its available bed to admit patients with other disease states or with other neurological emergencies.

Our findings were consistent with previous studies demonstrating that EVD placement for patients with sICH and signs or symptoms of elevated ICP is an important and timely intervention. In patients with sICH, EVD placement was associated with lower mortality^3^–^5^ and good functional independence.^4^ However, while further study is needed to confirm our observations, our study also suggests that shorter time interval to EVD placement in these critically ill patients was also associated with lower odds of death. In addition, reducing delay of transfer from the EDs was also associated with improved patient outcomes. A previous study showed that sICH patients who waited for more than five hours in the ED were associated with higher mortality.^8^ Further study is needed to investigate whether the CCRU, which was able to reduce delay of transfer when compared with transferring to traditional ICUs, would be associated with improved outcomes in patients with sICH.

## LIMITATIONS

Our study had several limitations. In this pre-post analysis, we were not able to account for possible changes of neurosurgeons’ practice regarding EVD placement. Its retrospective nature also prevented us from elucidating the medical decision-making processes regarding when to place EVD in these critically ill patients. Furthermore, mortality may not represent an effective outcome marker as most patients died from withdrawal of life support. We did not have 90-day functional outcome, and we did not collect the data retrospectively as it was shown to be unreliable.^18^ We did not account for patients who presented initially and who were transferred from another ED to the ED at our home institution. These patients may have had early neurosurgical interventions but still received care in the ED setting; thus, their outcomes may not be comparable to those who were transferred to an ICU as the CCRU or the NCCU. Furthermore, the small sample size of 35 patients who were admitted from our ED (Figure 1) may not provide a statistically meaningful comparison at this time. Our study did not examine the effect of the CCRU on outcome of patients with sICH but did not require EVD placement. Finally, the results from our study may not be generalizable due to factors such as intensivist shortage, costs, and different institutional needs. For example, the University of Michigan Emergency Critical Care Center was established to improve access to critical care for patients in its EDs,^19^ while the CCRU serves as a regional ICU.

## CONCLUSION

We demonstrated that the Critical Care Resuscitation Unit can complement the specialty Neurocritical Care Unit in the care of patients with sICH and who required EVD placement in the hyperacute and acute phase. The CCRU increased the overall numbers of patients with sICH requiring EVD placement who were transferred to our medical center from outlying EDs. Patients transferred to the CCRU had shorter transfer time than those admitted directly to the NCCU, although both the CCRU and the NCCU had similar time to EVD placement once the patients arrived at our medical center. Thus, a resuscitation unit can improve overall care for patients with spontaneous intracranial hemorrhage by reducing ED length of stay while facilitating urgent, time-saving procedures. Finally, delays in EVD placement in patients with spontaneous intracranial hemorrhage was associated with increased mortality.

## Figures and Tables

**Table 1A:** Characteristics of patients with spontaneous intracranial hemorrhage who were transferred from emergency departments to the critical care resuscitation unit or other intensive care units at a tertiary academic medical center.

	CCRU	Other ICUs		P-value	
		
	08/2013–09/2015) Group A	01/2011–07/2013 Group B	08/2013–09/2015 Group C	A versus B	A versus C
Total Patient n, (n per month)	123 (4.9)	81 (2.7)	55 (2.2)		
Age (years), mean (SD)	59 (14)	57 (14)	58 (14)	0.69	0.69
Gender					
Female, n (%)	70 (57)	48 (59)	32 (58)	0.84	0.99
Male, n (%)	53 (43)	33 (41)	23 (42)		
Ground distance (km), mean (SD)	28 (40)	33 (45)	26 (26)	0.47	0.47
Transport type, n (%)					
Ground	76 (62)	60 (74)	39 (71)	0.09	0.31
Air	47 (38)	21 (26)	16 (29)		
Intracranial hemorrhage type, n (%)					
IPH	34 (28)	28 (35)	38 (69)	0.37	<0.001
SAH	89 (72)	53 (65)	17 (31)		
Seizure, n (%)					
No	113 (91)	72 (89)	46 (84)	0.64	0.17
Yes	10 (9)	9 (11)	9 (16)		
Mechanical ventilation in ED, n (%)					
No	54 (44)	38 (47)	24 (44)	0.77	0.88
Yes	69 (56)	43 (53)	31 (56)		
Severity					
ESI**, median [IQR]	2 [1–3]	2 [2–3]	2 [1–3]	0.042	0.076
Hunt and Hess*	3 [2–4]	3 [2–4]	2 [2–3]	0.08	0.08
WFNSS*	4 [2–4]	4 [2–5]	2 [2–4]	0.29	0.29
ICH, mean (SD)*	3 (1)	2 (1)	3 (1)	0.10	0.10
FUNC**	3 (1)	8 (2)	6 (2)	0.07	0.07
Anticoagulation, n (%)	27 (22)	19 (23)	16 (29)	0.92	0.40
Anticoagulation, n(%)	11 (9)	6 (7)	4 (7)	0.88	0.78
Anti-Platelet, n (%)	16 (13)	13 (16)	12 (21)	0.69	0.20
Triage GCS, median [IQR]	13 [7–15]	14 [9–15]	14 [7–15]	0.43	0.43
Triage SBP (mm Hg), mean (SD)	179 (40)	178 (40)	173 (36)	0.61	0.61
ED Maximum SBP (mm Hg), mean (SD)	196 (36)	198 (38)	190 (40)	0.47	0.47
ED Minimum SBP (mm Hg), mean (SD)	136 (26)	137 (27)	135 (25)	0.89	0.89
ED LOS (min), median [IQR]	173 [121–236]	189 [147–314]	195 [137–257]	0.06	0.32
EDMV length (min), median [IQR]	85 [56–129]	126 [66–168]	92 [70–151]	0.07	0.07
ICU first GCS, median [IQR]	9 [6–14]	8 [5–14]	9 [7–14]	0.65	0.65
ICU SBP (mm Hg), mean (SD)	140 (21)	155 (29)	147 (22)	<0.001	0.17
Intracranial opening pressure (cm H_2_0), mean (SD)	21 (8)	22 (8)	23 (7)	0.28	0.28

*CCRU*, critical care resuscitation; *ICU*, intensive care unit; *km*, kilometer; *IPH*, intraparenchymal hemorrhage; *SAH*, subarachnoid hemorrhage; *ESI*, Emergency Severity Index; *WFNSS*, World Federation of Neurosurgeons Scale Score; *ICH*, Intracerebral Hemorrhage Score; *FUNC*, Functional Outcomes in Patients with Primary Intracerebral Hemorrhage score; *ED*, emergency department; *LOS*, length of stay; *EDMV*, mechanical ventilation in the emergency department; *GCS*, Glasgow Coma Scale; *SBP*, systolic blood pressure *IQR*, interquartile range; *cm H_2_0*, centimeters of water; *SD*, standard deviation; *mm Hg*, millimeters mercury.

**Table 1B:** Demographic characteristics of subgroups of patients with subarachnoid hemorrhage and intraparenchymal hemorrhage who were transferred from emergency departments to a tertiary care academic medical center during the study period.

	All patients	Only SAH	Only IPH	P-value (SAH versus IPH)
Total Patient (n)	259	180	79	
Age (years), mean (SD)	58 (14)	58 (13)	60 (14)	0.15
Gender				
Female, n (%)	150 (58)	117 (65)	33 (32)	0.001
Male, n (%)	109 (42)	63 (35)	46 (58)	
Ground distance (km), mean (SD)	29 (40)	28 (32)	33 (53)	0.42
Transport type, n (%)				
Ground	175 (68)	120 (67)	55 (70)	0.64
Air	84 (32)	60 (33)	24 (30)	
Seizure, n (%)	27 (10)	16 (9)	11 (14)	0.22
Mechanical ventilation in ED, n (%)	143 (55)	84 (47)	59 (75)	0.001
Disease severity				
ESI**, median [IQR]	2 [1–3]	2 [1–3]	2 [1–3]	0.21
Hunt and Hess*, median [IQR]	3 [2–4]	3 [2–4]	NA	NA
WFNSS*, median [IQR]	4 [2–4]	4 [2–4]	NA	NA
ICH, mean (SD)*	2.5 (1)	NA	2.5 (1)	NA
FUNC**	7 (2)	NA	7 (2)	NA
Anticoagulation, n (%)	21 (12)	10 (6)	11 (14)	0.02
Anti-platelet, n (%)	41 (23)	20 (11)	21 (26)	0.002
Triage GCS, median [IQR]	14 [7–15]	14 [10–15]	9 [6–14	0.001
Triage SBP (mm Hg),				
Mean (SD)	178 (39)	174 (35)	186 (44)	0.03
ED max SBP (mm Hg), mean (SD)	195 (37)	190 (36)	207 (39)	0.001
ED Min SBP (mm Hg), mean (SD)	136 (26)	135 (25)	138 (28)	0.36
ED LOS (min), median [IQR]	181 [134–262]	184 [130–267]	173 [142–253]	0.78
EDMV length (min), median [IQR]	100 [60–148]	87 [54–153]	105 [66–142]	0.34
ICU First GCS, median [IQR]	9 [6–14]	9 [6–14]	7 [6–12]	0.051
ICU SBP (mm Hg), mean (SD)	146 (25)	145 (23)	151 (27)	0.06
Intracranial Opening pressure (cm H_2_0), mean (SD)	21 (7)	22 (7)	21 (8)	0.36

* Higher score, higher severity

** Lower score, higher severity

*SAH*, subarachnoid hemorrhage; *IPH*, intraparenchymal hemorrhage; *km*, kilometer; *cm H_2_O*, centimeters of water; *ED*, emergency department; *EDMV*, mechanical ventilation in the emergency department; *ESI*, Emergency Severity Index; external ventricular drain; *FUNC*, Functional Outcome in Patients with Primary Intracerebral Hemorrhage score; *GCS*, Glasgow Coma Scale; *ICH*, Intracranial Hemorrhage score; *WFNSS*, World Federation of Neurological Surgeons scale; IQR, interquartile range; *LOS*, length of stay; min; minute; *SBP*, systolic blood pressure; *SD*, standard deviation; *mm Hg*, millimeters mercury.

**Table 2A:** Comparisons of time-to-event and hospital outcomes between patients who were transferred from emergency departments to the critical care resuscitation unit or other intensive care units.

	CCRU	Other ICUs		P-value	
		
	(08/2013–09/2015) (Group A) (N=123)	01/2011–07/2013 (Group B) (N=81)	08/2013–09/2015 (Group C) (N=55)	A vs B	A vs C
Arrival-EVD Placement (min), Median [IQR]	170 [106–311]	241 [152–491]	210 [139–574]	<0.01	0.28
Transfer Request-Arrival (min), median [IQR]	84 [61–111]	135 [89–255]	132 [99–177]	<0.001	<0.001
Hospital LOS (day), median [IQR]	20 [12–28]	22 [15–32]	21 [14–31]	0.47	0.47
Mortality, n (%)	31 (25)	14 (17)	14 (25)	0.23	0.88
Discharge Home, n (%)	24 (19)	20 (24)	13 (24)	0.48	0.67

*CCRU*, critical care intensive care unit; *ICU*, intensive care unit; *EVD*, external ventricular drain; *min*, minutes; *IQR*, interquartile ratio; *LOS*, length of stay.

**Table 2B:** Comparisons of time-to-event and hospital outcomes between patients who were transferred from emergency departments to a tertiary care center for management of either subarachnoid hemorrhage only or intraparenchymal hemorrhage only.

	All Patients (N= 259)	Only SAH (N=180)	Only IPH (N=79)	P-value (SAH vs IPH)
Arrival-EVD Placement (min), median [IQR]	203 [130–426]	202 [132–377]	224 [112–507]	0.63
Transfer Request - Arrival (min), median [IQR]	103 [76–155]	102 [70–152]	111 [86–162]	0.10
Hospital LOS (day), median [IQR]	20 [13–30]	20 [14–29]	22 [13–32]	0.33
Mortality, n (%)	59 (23)	37 (21)	22 (28)	0.20
Discharge Home, n (%)	57 (22)	50 (28)	7 (9)	0.001

*SAH*, subarachnoid hemorrhage; *IPH*, intraparenchymal hemorrhage; *EVD*, external ventricular drain; *min*, minutes; *IQR*, interquartile range; *LOS*, length of stay.

**Table 3:** Multivariable logistic regressions assessing association between clinical factors and mortality.

Variables	All patients			SAH only			IPH only		
		
	OR	95% CI	P-value	OR	95% CI	P-value	OR	95% CI	P-value
ARR-EVD#	**1.0003**	**1.0001–1.006**	**0.043**	**1.0001**	**1.0001–1.001**	**0.016**	1.0	1.00–1.001	0.29
Age	**1.04**	**1.008–1.066**	**0.012**	**1.04**	**1.005–1.08**	**0.024**	1.07	0.9–1.2	0.06
12-Hour GCS	**0.79**	**0.67–0.91**	**0.001**	**0.8**	**0.7–0.99**	**0.047**	**0.6**	**0.4–0.94**	**0.02**
CNSLT-ARR#	0.99	0.99–1.002	0.21	1.0	0.9–1.003	0.096	0.99	0.9–1.004	0.25
Admit-CCRU	0.8	0.4–2.3	0.92	0.86	0.3–2.2	0.77	0.9	0.9–1.004	0.91
EDMV	1.48	0.39–5.6	0.56	1.6	0.3–9.6	0.64	0.3	0.01–4.9	0.38
ESI**	1.02	0.6–1.7	0.93	1.2	0.6–2.5	0.52	1.3	0.5–3.3	0.65
Triage GCS	0.90	0.80–1.04	0.28	0.96	0.83–1.1	0.54	0.86	0.7–1.07	0.18
ED Lowest SBP	0.98	0.98–1.003	0.15	0.99	0.9–1.009	0.30	0.9	0.9–1.002	0.07
EDMV LOS	0.99	0.99–1.004	0.42	0.99	0.9–1.004	0.32	1.0	0.9–1.01	0.97
ICU GCS	0.93	0.82–1.06	0.29	0.94	0.8–1.01	0.43	0.9	0.6–1.2	0.56
Type of Hemorrhage ICH	0.86	0.39–1.9	0.72	NA	NA	NA	NA	NA	NA
Hunt and Hess*	NA	NA	NA	**2.8**	**1.3–6.2**	**0.008**	NA	NA	NA
WFNSS*	NA	NA	NA	0.6	0.2–1.4	0.23	NA	NA	NA
ICH*	NA	NA	NA	NA	NA	NA	1.9	0.7–5.0	0.19
FUNC**	NA	NA	NA	NA	NA	NA	1.5	0.8–2.6	0.20

# clinically significant factor

* Higher score, higher disease severity.

** Lower score, higher disease severity

NA Variable was not included in multivariable logistic regression.

Bolded variables were associated with statistical significance.

*SAH*, subsrachnoid hemorrhage; *IPC*, interparenchymal hemorrhage; *ARR-EVD*, time intervals in minutes between arrival at ICU and placement of external ventricular drain; *CNSLT-ARR*, time intervals in minutes between transfer request and arrival at ICU; *Admit-CCRU*, admission to the critical care resuscitation unit; *CI*, confidence interval; *ED*, emergency department; *EDMVLOS*, duration of invasive mechanical ventilation in minutes while in ED; *ESI*, Emergency Severity Index; *FUNC*, Functional Outcome in Patients with Primary Intracerebral Hemorrhage score; *GCS*, Glasgow Coma Scale; *ICH*, Intracerebral Hemorrhage score; *ICU*, intensive care unit; *WFNSS*, World Federation of Neurological Surgeon Scale.
